# Expression and Functional Heterogeneity of Chemokine Receptors CXCR4 and CXCR7 in Primary Patient-Derived Glioblastoma Cells

**DOI:** 10.1371/journal.pone.0059750

**Published:** 2013-03-21

**Authors:** Che Liu, Kien Pham, Defang Luo, Brent A. Reynolds, Parvinder Hothi, Gregory Foltz, Jeffrey K. Harrison

**Affiliations:** 1 Department of Pharmacology & Therapeutics, College of Medicine, University of Florida, Gainesville, Florida, United States of America; 2 Department of Neurosurgery, College of Medicine, University of Florida, Gainesville, Florida, United States of America; 3 The Ben and Catherine Ivy Center for Advanced Brain Tumor Treatment, Swedish Neuroscience Institute, Seattle, Washington, United States of America; University of Pécs Medical School, Hungary

## Abstract

Glioblastoma (GBM) is the most common primary brain tumor in adults. The poor prognosis and minimally successful treatments of these tumors indicates a need to identify new therapeutic targets. Therapy resistance of GBMs is attributed to heterogeneity of the glioblastoma due to genetic alterations and functional subpopulations. Chemokine receptors CXCR4 and CXCR7 play important roles in progression of various cancers although the specific functions of the CXCL12−CXCR4−CXCR7 axis in GBM are less characterized. In this study we examined the expression and function of CXCR4 and CXCR7 in four primary patient-derived GBM cell lines of the proliferative subclass, investigating their roles in in vitro growth, migration, sphere and tube formation. CXCR4 and CXCR7 cell surface expression was heterogeneous both between and within each cell line examined, which was not reflected by RT-PCR analysis. Variable percentages of CXCR4+CXCR7− (CXCR4 single positive), CXCR4−CXCR7+ (CXCR7 single positive), CXCR4+CXCR7+ (double positive), and CXCR4−CXCR7− (double negative) subpopulations were evident across the lines examined. A subpopulation of slow cell cycling cells was enriched in CXCR4 and CXCR7. CXCR4+, CXCR7+, and CXCR4+/CXCR7+ subpopulations were able to initiate intracranial tumors in vivo. CXCL12 stimulated in vitro cell growth, migration, sphere formation and tube formation in some lines and, depending on the response, the effects were mediated by either CXCR4 or CXCR7. Collectively, our results indicate a high level of heterogeneity in both the surface expression and functions of CXCR4 and CXCR7 in primary human GBM cells of the proliferative subclass. Should targeting of CXCR4 and CXCR7 provide clinical benefits to GBM patients, a personalized treatment approach should be considered given the differential expression and functions of these receptors in GBM.

## Introduction

Human glioblastoma (GBM), classified grade IV according to WHO, is the most malignant form of primary brain tumor in adult humans. Current treatment paradigms for GBM are surgical resection of the tumor mass, followed by adjuvant radiotherapy and chemotherapy. Unfortunately, these approaches only modestly improve the survival rate of GBM patients. A major reason why GBMs are resistant to therapies is because of a high degree of cellular and molecular heterogeneity. GBM consists of cells that are genetically and physiologically different from each other. Due to the highly heterogeneous nature of GBM, studies are focusing on identifying genetic alterations and molecular pathways associated with subclasses of GBMs [1], [2], [3], [4], [5]. Four molecular subclasses of GBMs, including classic, neural, proneural, and mesenchymal, have been determined according to their genetic alterations and gene expression profiles [4]. A prior classification by Phillips et al. identified three subclasses, termed proneural, mesenchymal, and proliferative [3]. Molecular based classifications of GBMs provide a more precise tool in patient prognosis. In addition, identification of novel therapeutic targets in individual molecular subclasses is critical in order to improve the effectiveness of treatments. However, these molecular subclasses are defined by genetic assays and therefore do not reflect potential heterogeneities resulting from post-transcriptional- and/or post-translational modifications of expressed proteins.

CXCR4 is a member of the CXC chemokine receptor sub-family and has a single endogenous ligand CXCL12 (SDF-1). CXCR4 and CXCL12 are one of the most well studied chemokine systems in tumor growth, metastasis, and angiogenesis. CXCR4 and/or CXCL12 are up-regulated in pancreatic cancer [6], colon cancer [7], ovarian cancer [8], lymphoma [9], medulloblastoma [10] and glioma [11], which suggests a critical role of CXCR4 in these cancers. CXCL12 is also constitutively expressed in tissues such as liver, lung, lymph nodes, adrenal glands and bone marrow, which indicates the important role of CXCL12/CXCR4 in tumor metastasis toward distant locations [12]. Indeed, inhibition of the CXCL12/CXCR4 axis decreases the metastasis of osteosarcoma and melanoma [13]. In the context of glioma, CXCR4 is elevated in GBM and grade III gliomas compared with grade II gliomas [14]. Antagonism of CXCR4 can inhibit human glioma growth [15], [16], [17], invasion [15], [17], and pro-MMP2 activation [17]. Several studies have shown that CXCL12 induces the migration, proliferation, capillary tube formation as well as VEGF production in endothelial cells [18], [19]. Furthermore, inhibition of CXCL12 and CXCR4 reduces tumor growth by blocking angiogenesis [20].

In addition to CXCR4, CXCL12 also interacts with an additional chemokine receptor termed CXCR7 [21] which can also bind to CXCL11 [21]. CXCR7 is expressed by a variety of cancers, including breast cancer [22], lung cancer [23], and glioma [24], [25]. Breast cancer lines stably over-expressing CXCR7 form larger tumors while other lines with CXCR7 silencing show decreased tumor volumes [23]. In lung cancer, CXCR7 not only promotes tumor growth but also enhances tumor metastasis [23]. Several studies suggest that CXCR7 contributes to tumor progression indirectly via regulation of CXCR4-dependent activities. For instance, CXCR7 regulates acute CXCR4 activation by depleting extracellular CXCL12 via CXCR7 internalization [22], [26]. CXCR7 exerts direct effects as a functional receptor, inducing cell adhesion of malignant hematopoietic cells through ERK 1/2 and AKT pathway activation [27]. In glioma, CXCR7 exhibits anti-apoptotic activity and thus promotes glioma tumor growth [25]. Therefore, the CXCL12−CXCR4−CXCR7 axis in cancers could be more complicated and the balance of direct and indirect activities of CXCR7 may play critical roles in tumor progression.

In this study, we investigated the roles of CXCR4 and CXCR7 in glioblastoma using primary patient-derived GBM cells. We found that CXCR4 and CXCR7 were heterogeneously expressed by GBM cells, in the proliferative subclass, on the cell surface despite similar levels of CXCR4 and CXCR7 mRNAs. The slow cycling subpopulation in GBM cells, which is enriched with cancer stem cell markers, showed increased levels of cell surface CXCR4 and CXCR7. Functional characterization of CXCR4 and CXCR7 revealed diverse roles of both receptors in promoting tumor cell growth, migration, sphere formation, and tube formation in vitro and depended on the specific GBM line. Collectively, our results indicate that CXCR4 and CXCR7 are involved in important activities associated with glioblastoma progression. Together with CXCR4, CXCR7 is a potential therapeutic target, to be considered for individualized treatments that depend on the specific GBM patients.

## Materials and Methods

### Cell Culture

Primary patient-derived GBM cell lines L0, L1, L2 [28] were isolated by Dr. Brent Reynolds at University of Florida, Gainesville, FL, while GBM cell line S2 [29] was generated by Drs. Parvinder Hothi and Gregory Foltz at Swedish Neuroscience Institute, Seattle, Washington. All the cells were maintained in DMEM/F12 medium supplemented with 2% B27, 1% penicillin-streptomycin, 20 ng/ml human EGF, and 10 ng/ml human bFGF. All the cells were grown in a humidified incubator at 37°C with 5% CO_2_. DMEM/F12 medium, B27, EGF, bFGF, and antibiotics were obtained from Gibco (Life Technologies, CA). Cells were used within the following range of passage numbers: L0 (88–98); L1 (4–14); L2 (14–24); S2 (4–14).

### Animals

NOD-scid IL2Rγ^null^ (NSG) mice were obtained from Jackson Laboratories, ME. All procedures involving mice were carried out in accordance with the guidelines of, and were specifically approved by, the University of Florida Institutional Animal Care and Use Committee (IACUC).

### Reverse Transcription-Polymerase Chain Reaction (RT-PCR)

Total RNA was isolated from GBM cells with the TRIzol reagent (Invitrogen, Life Technologies, CA) according to the manufacturer’s instructions. Genomic DNA contamination was removed by RQ1 RNase-free DNase treatment (Promega, WI). Total RNA was then quantified and stored at −80°C. RNA (1 µg) was retrotranscribed with iScript cDNA synthesis kit (BioRad, CA). Synthesized cDNA was subjected to PCR analysis. Touchdown PCR was performed by heating for 96°C for 2 min, followed by the touchdown phase for 15 cycles: 96°C for 30 sec, 65°C for 1 min (decrease the annealing temperature by 1°C per cycle), and 72°C for 1 min. After touchdown phase is the amplification stage for 30 cycles: 96°C for 30 sec, 50°C for 1 min, and 72°C for 1 min. The following primers were used: human CXCL11∶5′-CCTGGGGTAAAAGCAGTGAA-3′ (forward), and 5′-TGGGGAAAGAAGTGTGTATTTG-3′ (reverse); human CXCL12∶5′-AGAGCCAACGTCAAGCATCT-3′ (forward), and 5′-AGGGTCTAAATGCTGGCAAA-3′ (reverse); human CXCR4∶5′-GGCCCTCAAGACCACAGTCA-3′ (forward), and 5′-TTAGCTGGAGTGAAAACTTGAAG-3′ (reverse); human CXCR7∶5′-GCAGAGCTCACAGTTGTTGC-3′ (forward), and 5′-CCGGCAGTAGGTCTCATTGT-3′ (reverse); human actin: 5′-CTCTTCCAGCCTTCCTTCCT-3′ (forward) and 5′-CACCTTCACCGTTCCAGTTT-3′ (reverse).

### Intracranial Injection of GBM Cells

For implantation, GBM cells (5×10^5^) in 1 µl were injected 3 mm deep into the right cerebral hemisphere (1 mm posterior and 2 mm lateral from bregma) of NSG mice. The endpoint was defined by a lack of physical activity and a body weight reduction of greater than 15%. Tumor-bearing mice were euthanized using sodium pentobarbital (32 mg/kg) and subsequently perfused with 0.9% saline followed by buffered 4% paraformaldehyde (PFA). Brains were surgically removed and post-fixed with 4% PFA. After fixation, tissues were incubated in 30% sucrose solution at 4°C overnight followed by either liquid nitrogen freezing or paraffin-embedding. Brains were then sectioned and subjected to immunohistochemistry.

### Immunohistochemistry

For immunohistochemistry, the slides were heated for 30 min at 70°C, deparaffinized in Xylene (3 times, 5 min each), and then rehydrated by stepwise immersion in 100% EtOH (2×5 min), 95% EtOH (2×5 min), and 70% EtOH (1×3 min). After deparaffinization, the samples were rinsed with deionized water and processed to antigen retrieval with sodium citrate buffer, pH 6, for 20 min at 98°C. After cooled down to room temperature, the samples were washed with deionized water for 5 min, quenched with 3% H_2_O_2_ for 10 min at room temperature to block endogenous peroxidase activities, and processed to standard immunohistochemistry staining. Briefly, the sections were initially blocked with 1% BSA in TBS-T for 30 min and then incubated in mouse anti-human nestin antibody (1∶100, Thermo Fisher Scientific, MA) at room temperature. After 2 hr, sections were washed three times with TBS-T and incubated with secondary goat anti-mouse HRP antibody for 1 hr at room temperature. The tissues were then washed three times with TBS-T and developed with 3,3′-Diaminobenzidine (DAB) kit per manufacturer’s instructions (Vector Labs, CA). Samples were counterstained with hematoxylin.

### CarboxyFluorescein Succinimidyl Ester (CFSE) Staining

To identify the slow cycling subpopulation, primary patient-derived GBM cells were loaded with CellTrace CFSE green fluorescent dye (Molecular Probes, Life Technologies, NY) according to the manufacturer’s instructions. In short, cells were incubated with 5 µM CFSE in PBS for 10 min. Reaction was terminated by adding equal volume of medium, washed, and cells were cultured subsequently for 7 days. Slow-cycling cells, defined as top 5% CFSE intensity, and overall population (bottom 85%) were determined as previously described [28] and were analyzed 7 days after CFSE staining.

### Flow Cytometry and Fluorescence-activated Cell Sorting (FACS)

Cells were harvested with 0.02% EDTA in PBS, pH 7.4, washed with ice cold 1% BSA in PBS and subsequently blocked with 5 µg/ml of mouse IgG for 15 min at room temperature. Cells were then incubated with specific antibody for 30 min on ice. Mouse anti-human CXCR4-PE, mouse-anti human CXCR7-APC, mouse-anti human CXCR3-PE, and mouse anti-human CCR3-APC (dilution 1∶20, R&D Systems, MN) were used. Samples were then washed and analyzed with BD LSR II system (BD Biosciences, CA). Parallel samples were analyzed with the respective PE- or APC-conjugated non-immune IgGs to determine non-specific staining and determination of quadrants in flow cytometry plots. Dead cells were excluded by 4′,6-diamidino-2-phenylindole (DAPI, Sigma-Aldrich, MO) staining. All data were analyzed by FlowJo software version 7.6 (Tree Star, OR).

### Proliferation Assay

Cells were plated in 48-well plates at a density of 10 cells/µl and treated with multiple concentrations of either CXCL11 or CXCL12 (R&D Systems, MN) at concentrations of 0.3 nM, 1 nM, 3 nM, 10 nM, 30 nM; 2 ng/ml of EGF was included in all conditions. Cells cultured in medium with 2 ng/ml EGF served as the control. Cell numbers were determined one week later. To determine the contribution of CXCR4 or CXCR7 stimulation of cell proliferation, cells cultured as described above were treated with 3 nM of CXCL12 combined with either 1 µM of AMD3100 (Sigma-Aldrich, MO) or 100 nM of CCX733, CCX771, or CCX704 (Chemocentryx, CA). All experiments were performed in triplicate and are representative of three independent experiments.

### Apoptosis Assay

Cells were plated in 6-well plates at a density of 5×10^4^ cells/ml at day 0 in DMEM/F12 medium supplemented with 2% B27, 1% penicillin-streptomycin, 20 ng/ml human EGF, and 10 ng/ml human bFGF. To induce apoptosis, temozolomide at a concentration of 500 µM (Sigma-Aldrich, MO) was added to the sphere cultures at day 4, 5, and 6, in the presence and absence of CXCL12 (5 nM). At day 7, cells were collected, stained with a cell-permeable, FITC-conjugated irreversible pan-caspase inhibitor (ApoStat, R&D Systems, Minneapolis, MN) according to the manufacturer’s instructions, and subjected to flow cytometry analysis to quantitate caspase activity of cells undergoing apoptosis. Unstained samples were included as controls to determine nonspecific staining in each treated group.

### Migration Assay

GBM cells were dissociated with 0.02% EDTA in PBS, pH7.4, counted, and 10^5^ cells were transferred to 8-µm pore size cell culture inserts (BD Bioscience, CA) in growth factor-free medium and the assembly placed into 24-well plates containing multiple concentrations of either CXCL11 or CXCL12 (0.3 nM, 1 nM, 3 nM, 10 nM, 30 nM). After 6 h, migrating cells in the bottom well were fixed with 4% paraformaldehyde, stained with DAPI, and counted. A combination of 3 nM of CXCL12 with 1 µM of AMD3100 or 100 nM of the compounds CCX733, CCX771, or CCX704 were performed to determine the effect of CXCR4 and CXCR7 inhibition on cell migration.

### Primary Sphere Formation Assay

Primary sphere formation assays were performed to quantify stem-like cell frequency within primary GBM cells with CXCL12 stimulation. Cells were plated in 384-well plates at a density of 500 cells/50 µl/well containing multiple concentrations of either CXCL11 or CXCL12 (0.3 nM, 1 nM, 3 nM, 10 nM, 30 nM). After 7 days, sphere numbers were counted. Combination of 10 nM of CXCL12 with 1 µM of AMD3100 or 100 nM of CCX733, respectively were performed to determine the effect of CXCR4 and CXCR7 inhibition on sphere formation.

### Tube Formation Assay

Cells were dispersed into single cells and cultured in Matrigel (BD Biosciences, CA) coated 48 well-plates, at a density of 45,000 cells/well, with serum-free M131 medium supplemented with 5% of microvascular growth supplement (Invitrogen, Life Technologies, CA). The cells were treated with 20 nM of CXCL12, with or without 1 µM of AMD3100, or 100 nM of CCX733, CCX771, or CCX704. After 48 h, cells were stained with 2 µg/ml of AM Calcein fluorescent dye (BD Biosciences, CA) and photographed with Zeiss inverted microscope. Total tube length, tube area, and branch points were measured using Metamorph software.

### Statistical Analysis

All statistical analyses were calculated using either Microsoft Excel or GraphPad Prism 5 software (GraphPad Software, Inc., CA). All data are presented as mean ± S.E.M. P values were calculated using Student’s t test with two-tailed distribution. A P value under 0.05 was considered significant and is indicated with asterisks in figures.

## Results

### Heterogeneous Cell Surface Expression of CXCR4 and CXCR7 by Primary Patient-derived GBM Cells

**Figure 1:**
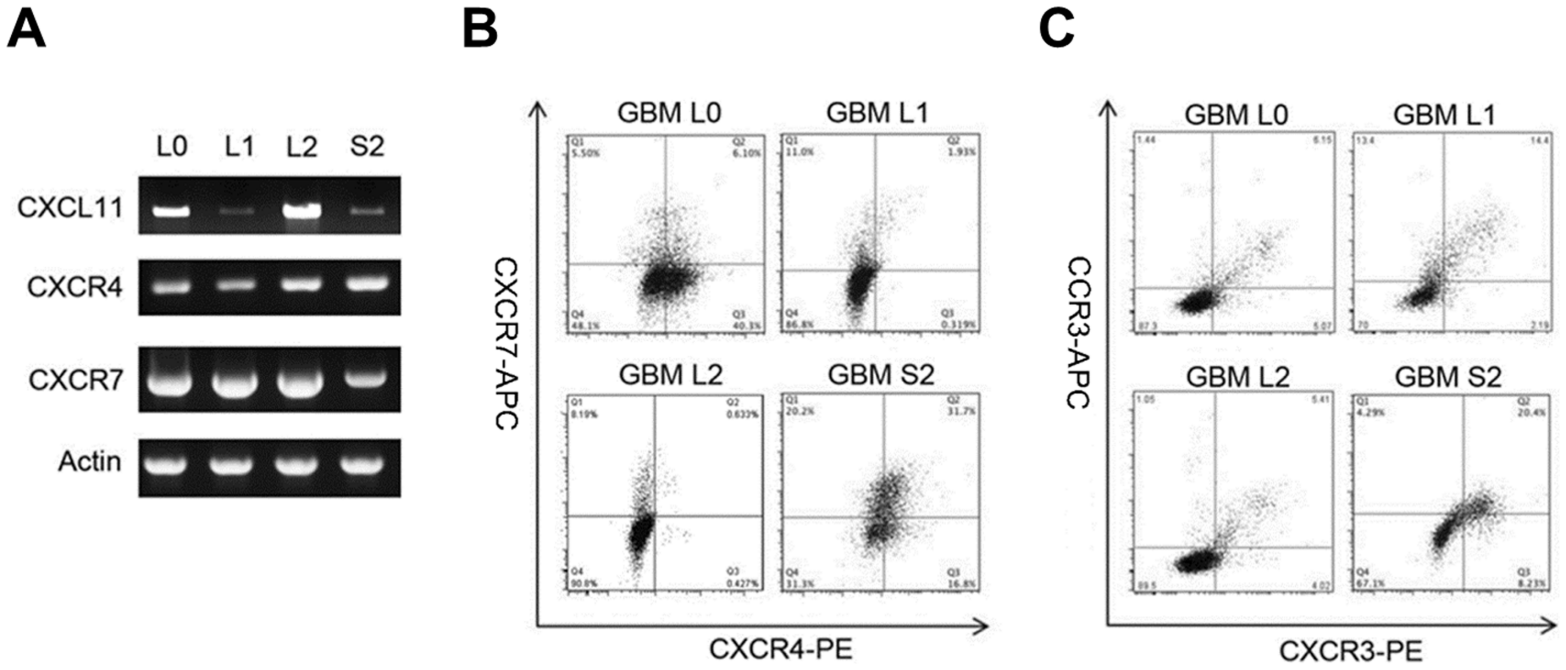
CXCL11, CXCR4, and CXCR7 expression by primary patient-derived GBM cells. (A) RT-PCR analysis detected CXCL11, CXCR4, and CXCR7 mRNAs in L0, L1, L2, and S2 cells. Actin was used as a control. Panels are representative from three independent experiments. (B) CXCR4 and CXCR7 are heterogeneously expressed on the cell surface of primary GBM cells as determined by flow cytometry analysis. Representative pictures are shown. (C) CCR3 and CXCR3 are co-expressed on the cell surface of primary GBM cells as determined by flow cytometry analysis. Representative pictures are shown.

The expression of CXCR4 and CXCR7 in glioma cell lines were documented previously and CXCR7 is up-regulated in high grade gliomas [25]. This prompted us to elucidate the roles of CXCR4 and CXCR7 in GBM progression utilizing primary GBM cells derived from four different patients, namely GBM L0, L1, L2, and S2; all lines were characterized to be in the proliferative subclass [3]. Levels of CXCL11, CXCL12, CXCR4, and CXCR7 mRNAs were determined by subjecting cell extracts to RT-PCR analysis while cell surface protein expression of the receptors were determined using flow cytometry. CXCR4 and CXCR7 mRNAs were expressed by all primary GBM cell lines (Figure 1A). The levels of CXCR4 and CXCR7 mRNAs were comparable across all the lines, although CXCR7 mRNA was somewhat lower in the S2 line (Figure 1A). CXCL11 mRNA was also present but variable in all lines with high (L2), intermediate (L0) and low (L1, S2) levels (Figure 1A). CXCL12 mRNA was undetectable in all cell lines examined (data not shown). Unlike the mRNA expression, CXCR4 and CXCR7 proteins on the cell surface showed heterogeneous expression (Figure 1B). All lines can be divided into CXCR4+CXCR7+, CXCR4+CXCR7−, CXCR4−CXCR7+, and CXCR4−CXCR7− according to their surface levels of both receptors. GBM L0 had a relatively higher percentage of CXCR4-expressing cells than CXCR7-expressing cells. In contrast, GBM L1 and L2 consisted of relatively more CXCR7-positive cells than CXCR4-positive cells. GBM S2 showed comparable percentages of CXCR4- and CXCR7-expressing cells. GBM lines also expressed CXCR3 and CCR3; each line had a similar small sub-population of cells that co-expressed both of these chemokine receptors (Figure 1C).

### Enrichment of CXCR4 and CXCR7 in the Slow-cycling Subpopulation

**Figure 2:**
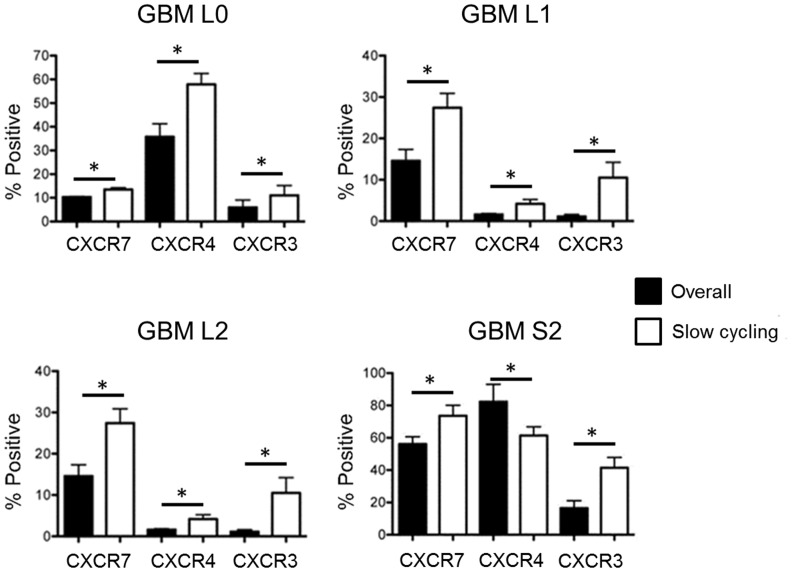
Increased CXCR3, CXCR4, and CXCR7 in slow cycling GBM cells. Flow cytometry identified significantly higher percentages of CXCR4-, CXCR7- and CXCR3-expressing cells in slow cycling subpopulations of GBM cells when compared to the overall population, with the exception of CXCR4-expressing cells in the overall population of S2 that is more abundant than in the slow cycling population. Filled bar: overall population. Open bar: slow cycling population. *P<0.05; **P<0.01.

The presence of a dye-retaining slow cycling subpopulation in primary patient-derived GBM cells that consists of a higher frequency of cancer stem cells and has greater tumor-initiating capacity in vivo has been reported [28]. To determine if CXCR4 and CXCR7 are associated with this subpopulation, the expression of CXCR4 and CXCR7 in slow-cycling GBM cells was analyzed by flow cytometry. All of the primary GBM lines examined had higher percentage of CXCR7-expressing cells in slow-cycling population when compared with the percentage in overall population (Figure 2). In addition, an increased percentage of CXCR4-expressing cells in the slow-cycling population was evident in all primary GBM cells with the exception of the S2 line, which showed a slightly lower level of CXCR4-expressing cells in slow-cycling population than in the overall population (Figure 2). Slow cycling cells were also enriched in CXCR3 (Figure 2).

### CXCL12 Promoted in vitro Sphere Formation of GBM L0 Cells

**Figure 3:**
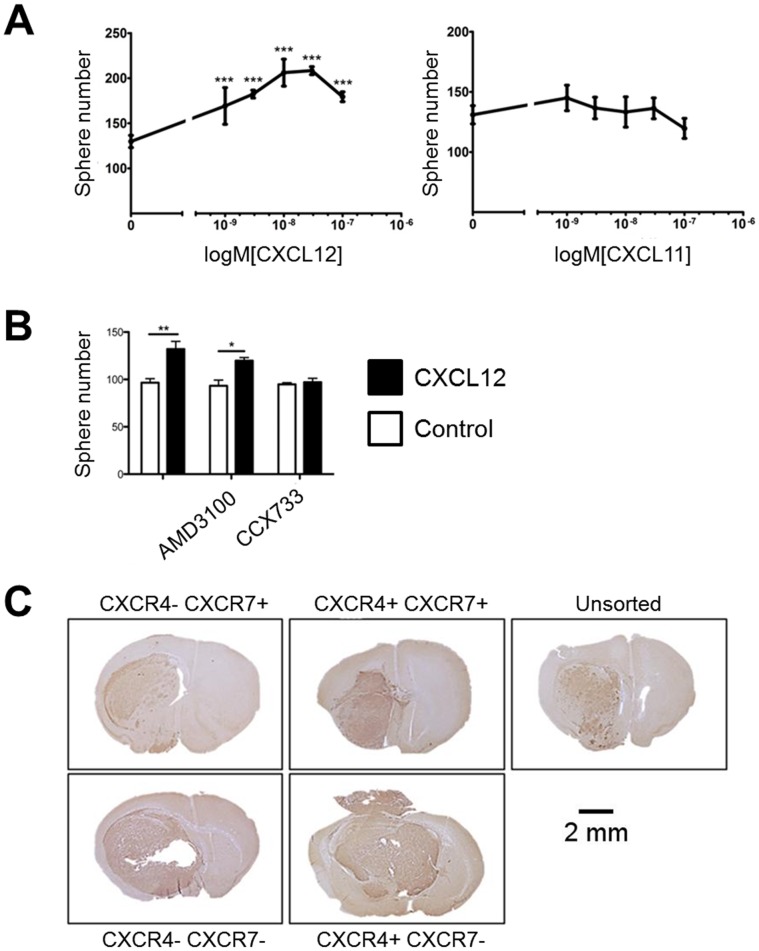
CXCL12 stimulated sphere formation in vitro and CXCR4+, CXCR7+, and CXCR4+/CXCR7+ cells generate tumors in vivo. (A) CXCL12 concentration-response assessment indicated that CXCL12 promoted in vitro sphere formation of L0 cells (0.3, 1, 3, 10, 30 nM, P<0.05). CCX733 (100 nM) suppressed CXCL12-regulated (10 nM) sphere formation while AMD3100 (1 µM) had no effect. All conditions contained 0.1% DMSO. ***P<0.001. (B) Representative sections from tumors derived from L0 sub-populations. The various sub-populations indicated in the figures were implanted intracranially into NSG mice. Shown are representative sections subjected to anti-human Nestin immunohistochemistry. Note that all sub-populations are capable of forming tumors in vivo.

Since CXCR4- and CXCR7-positive cells were more abundant in the slow-cycling subpopulation in a similar manner to what has been shown with other cancer-stem cell markers, we hypothesized that CXCR4 and/or CXCR7 might regulate cancer stem cell phenotypes. To test this hypothesis, we addressed if CXCL12 stimulation could impact *in vitro* sphere formation via CXCR4 and/or CXCR7. Our data indicated that CXCL12 promoted sphere formation of GBM L0 but had no effect on the other lines (Figure 3A); CXCL11 was without effect. CXCR7 antagonists CCX733 blocked CXCL12-induced sphere formation of L0 while the CXCR4 antagonist AMD3100 had no effect (Figure 3A). In the presence of 2 ng/ml of EGF, CXCL12 had no effect on L0 sphere formation (data not shown). These results suggested that CXCR7, but not CXCR4, is involved in sphere formation of GBM L0.

While in vitro sphere formation is a characteristic of tumor-initiating cells, tumor initiation in vivo is a more direct measure of this phenotype. To evaluate the in vivo tumor forming ability of CXCR4 and CXCR7-expressing cells, a single line was chosen to evaluate tumor formation of the various sub-populations of cells, i.e. the CXCR4+, CXCR7+, CXCR4+/CXCR7+ (double positive), and CXCR4−/CXCR7− (double negative) sub-populations. GBM L0 cells were chosen since this line showed stimulation of sphere formation by CXCL12. L0 cells were subjected to FACS and sub-populations based on CXCR4 or CXCR7 expression patterns were isolated and subsequently implanted intracranially in immune-deficient NSG mice. All sub-populations successfully established tumors in vivo (Figure 3C).

### CXCL12 Regulated Cell Proliferation and Migration via CXCR4 and CXCR7 in Human GBM Cells

**Figure 4:**
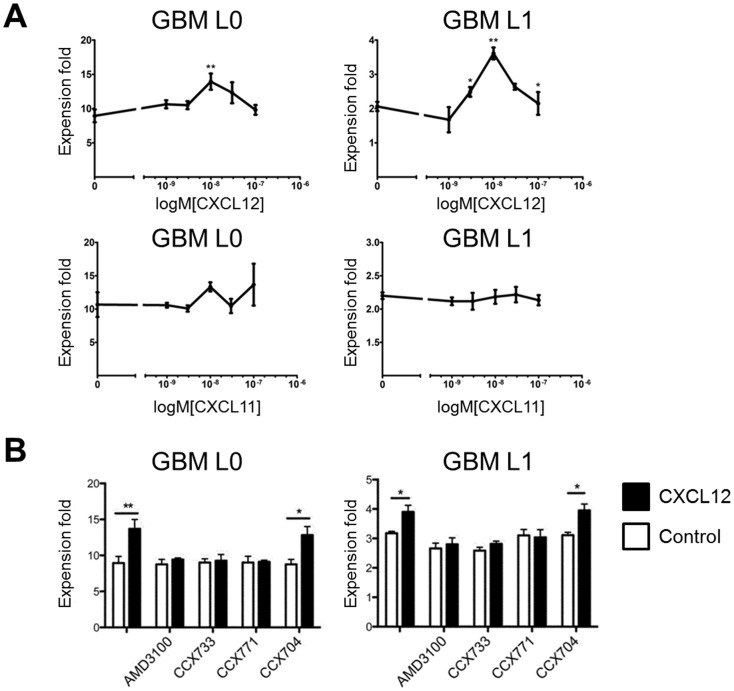
CXCL12 promoted cell growth through both CXCR4 and CXCR7. (A) CXCL12 concentration-response assessment showed that CXCL12 significantly enhanced cell growth in L0 and L1 cells. *P<0.05; **P<0.01. Representative results of three individual experiments performed in triplicate are shown. (B) AMD3100 (1 µM), CCX733 (100 nM), and CCX771 (100 nM) significantly blocked CXCL12 (10 nM) induced cell growth in L0 and L1 cells. CCX704 (100 nM) had no effect. All conditions contained 0.1% DMSO. *P<0.05; **P<0.01. Representative results of three individual experiments performed in triplicate are shown.

To address the functions of CXCR4 and CXCR7 in these primary GBM cells, we evaluated the effect of CXCL12 stimulation on in vitro cell growth. CXCL12 promoted cell proliferation of L0 and L1 in a dose dependent manner (Figure 4A) while CXCL11 did not impact in vitro growth of either line. The cell growth effect was inhibited by CXCR4 antagonist AMD3100 and two CXCR7 antagonists CCX733 and CCX771 (Figure 4B). CCX704, an inactive analog of CCX733, did not diminish CXCL12-regulated cell growth (Figure 4B). CXCL12 did not induce cell proliferation of either L2 or S2 cells.

**Figure 5:**
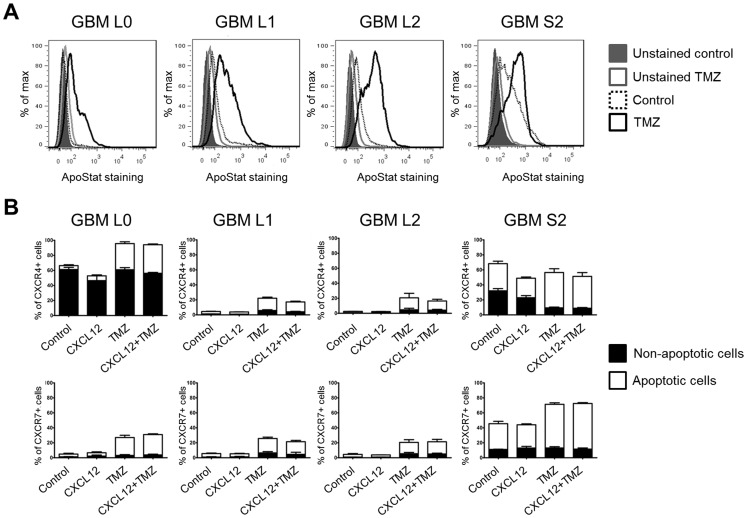
Differential impact of TMZ on the distribution of CXCR4- and CXCR7-expressing apoptotic and non-apoptotic subpopulations. (A) GBM lines were treated with TMZ as described in Methods. Apoptotic subpopulations, determined by positive ApoStat staining, were detected in TMZ treated samples in all cell lines. Histograms are representative from three independent experiments. (B) Heterogeneity in CXCR4- and CXCR7-expressing subpopulations in the apoptotic and non-apoptotic subpopulations after TMZ treatment, as determined by flow cytometry analysis. Bar graphs represent the average of three independent experiments. Data were analyzed with Graphpad Prism 5 software.

An anti-apoptotic effect mediated by stimulation of CXCR7, but not CXCR4, has been reported in GBM cell lines [25]. We evaluated the impact of CXCL12 on temozolomide (TMZ)-induced apoptosis of the various primary GBM cell lines. TMZ induced apoptosis in all four lines (Figure 5A). A differential effect of TMZ treatment on the relative percentages of CXCR4- and CXCR7-expressing subpopulations was evident. The percentage of CXCR4+ cells was increased in lines L0, L1, and L2 after TMZ treatment. TMZ did not impact the percentage of CXCR4-expressing supopulation in the S2 line. CXCR7+ subpopulations were increased in all lines after TMZ. Morevover, heterogeneous expression of CXCR4 and CXCR7 was observed between apoptotic and non-apoptotic subpopulations among the various cell lines after TMZ treatment. The percentage of CXCR4-expressing cells in the non-apoptotic population remained unchanged in L0 cells, increased in the L1 and L2 cells, and decreased in the S2 line. The percentage of CXCR7-expressing cells undergoing apoptois increased in all lines. Increases in the CXCR7+ subpopulation in the non apoptotic subpopulation occurred in the L0, L1, and L2 cells but not in the S2 line after TMZ treatment. In all lines, CXCL12 had no effect on the distribution of CXCR4- and CXZCR7-expressing cells in either the apoptotic or non-apoptotic populations.

**Figure 6:**
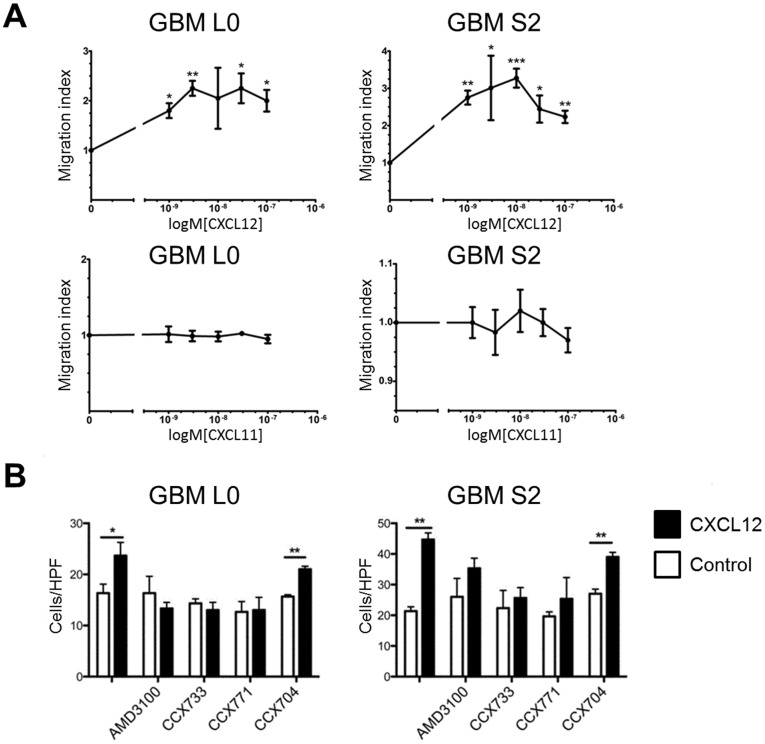
CXCL12 stimulated GBM cell migration in L0 and S2 cells is mediated by CXCR4 and CXCR7. (A) CXCL12 concentration-response assessment showed that CXCL12 significantly enhanced cell migration in L0 and S2 cells (0.3, 1, 3, 10, 30 nM). *P<0.05; **P<0.01. Representative results of three individual experiments performed in triplicate are shown. (B) AMD3100 (1 µM), CCX733 (100 nM), and CCX771 (100 nM) significantly blocked CXCL12 (3 nM) induced cell migration in L0 and S2 cells. *P<0.05; **P<0.01. CCX704 did not alter CXCL12 induced cell migration. All conditions contained 0.1% DMSO. Representative results of three individual experiments performed in triplicate are shown.

CXCR4 and CXCR7 ligands were also evaluated for effects on migration of GBM cells. CXCL12, but not CXCL11, significantly induced cell migration of L0 and S2 cells (Figure 6A). The migratory effect of CXCL12 was attenuated by blockade of either CXCR4 or CXCR7 (Figure 6B). CCX704 had no effect on CXCL12-mediated migration. Migration of L1 and L2 cells was not stimulated by CXCL11 or CXCL12 (data not shown).

### CXCL12 Induced Tube Formation of GBM L0 Cells via CXCR4

**Figure 7:**
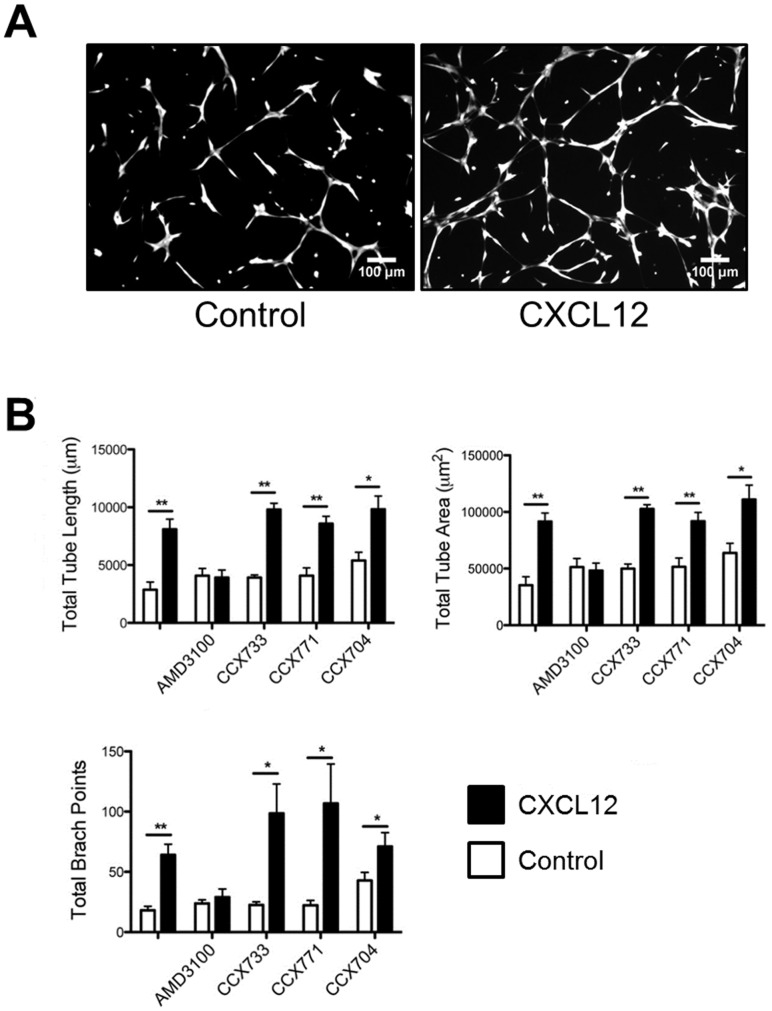
CXCL12 stimulation of L0 tube formation in vitro is mediated by CXCR4. (A) Representative images of control and CXCL12 (20 nM) treated GBM L0 cells. (B) CXCL12 (20 nM) significantly increased total tube length, total tube area, and total branch points of L0 cells. AMD3100 (1 µM) inhibited CXCL12 stimulation of tube formation of L0 cells. CXCR7 inhibitors did not block the stimulation of CXCL12. All conditions contained 0.1% DMSO. *P<0.05; **P<0.01. Representative results of three individual experiments performed in triplicate are shown.

Transdifferentiation of GBM tumor-derived endothelial cells has been documented by multiple groups [30], [31], [32] and CXCL12 has been reported to promote in vitro tube formation and VEGF production of endothelial cells [18], [19]. Therefore, we tested the hypothesis that CXCL12 might be involved in phenotypic changes associated with the differentiation of GBM cells into endothelial cells. CXCL12 promoted tube formation of GBM L0 cells (Figure 7A). Total tube area, total tube length, and total tube branch points were significantly increased by CXCL12 when compared to control treated L0 cells (Figure 7B). The effects of CXCL12 were blocked by AMD3100 but not CCX733 or CCX771, indicating that CXCR4, but not CXCR7, is involved in the CXCL12 stimulated-tube formation of L0 cells. CXCL12 had no effect on tube formation of the L1, L2, and S2 lines (data not shown).

## Discussion

GBMs are highly heterogeneous tumors, which consist of cells with different genetic and physiologic identities. Because of the heterogeneous nature of GBM, current treatment paradigms only exert modest impact on the survival of GBM patients. For example, temozolomide, an oral alkylating agent, only benefits GBM patients with epigenetic silencing of MGMT by promoter methylation [33]. Thus, the 54% of GBM samples examined that have unmethylated MGMT promoter [33] would require specific agents other than temozolomide. The fundamental drive to discover new therapies for these diverse GBMs has prompted studies that focused on identifying genetic alterations and molecular pathways associated with subclasses of GBMs [1], [2], [3], [4]. Currently, four molecular subclasses of GBMs are defined by either abnormalities in PDGFRA and IDH1 (proneural), EGFR (classic), NF1 (mesenchymal), or neuronal marker expression (neural) [4]. Other classification schemes, based on expression of markers associated with outcomes, include proneural, proliferative, and mesenchymal, and the relationship of these three subclasses with the more recent subclassification has been discussed [5]. Investigations of tumor-driven mechanisms in individual molecular subclasses are critical for development of personalized treatments for GBM patients. To this end, responsiveness to aggressive therapies is associated with the classic and mesenchymal subclasses, while the proneural subclass does not fare well with current treatments. However, a potential major issue of genetic or genomic-defined subclasses is that they do not reflect potential heterogeneities resulting from post-transcriptional- and/or post-translational modifications of expressed proteins. In this study, we analyzed four primary patient-derived GBM cell lines of the proliferative subclass [3]. All lines showed comparable levels of CXCR4 mRNA and lacked CXCL12 mRNA expression, which matched a GBM subtype defined by Schulte et al [2]. However, the cell surface expression of CXCR4 protein is highly heterogeneous amongst these lines. Similarly, CXCR7 also has discordant expression patterns when comparing mRNA and cell surface protein in the GBM cells. Heterogeneity was not associated with surface expression of CXCR3 and CCR3 which suggests that these phenotypic variations of CXCR4 and CXCR7 are not a general phenomena for chemokine receptors expressed on GBM cells. Previously, the association between GBMs and human cytomegalovirus (HCMV) has been documented [34], [35], [36]. Interestingly, HCMV proteins UL33 and UL78 have been shown to modulate surface CXCR4 level without altering total CXCR4 expression in leukemia cells in vitro [37]. This might be one of the explanations for the differential surface CXCR4 in GBM cells. In addition, it has been reported that the surface protein level of CXCR7 is not correlated with mRNA levels [21], which is consistent with our findings. Taken together, our study indicates a greater level of heterogeneity of chemokine receptors proteins CXCR4 and CXCR7 on human GBM cells than previously appreciated.

Our study also documents heterogeneity in functional responses to CXCL12 stimulation in GBM cells. Both CXCR4 and CXCR7 are involved in GBM cell growth and migration induced by CXCL12, a conclusion that is supported by blockade of these responses with antagonists for either receptor. However, the CXCL12-mediated cell growth and migration are not universal amongst the primary patient-derived GBM cells examined since only two out of four lines (L0 and L1) significantly responded to CXCL12 in terms of cell growth, while only two of the lines (L0 and S2) migrated toward CXCL12. Furthermore, CXCL12 significantly promoted in vitro sphere formation through CXCR7 and stimulation of in vitro tube formation through CXCR4 is evident only with the L0 cells. These data suggest that the downstream cellular events of CXCR4 and/or CXCR7 might be distinct in each GBM line, which indicates an even greater level of functional diversity associated with CXCL12 stimulation. Indeed, documented studies have shown that the CXCL12-CXCR4-CXCR7 axis is complex, as CXCR4 and CXCR7 can activate different downstream cellular events respectively after CXCL12 stimulation [38], [39], [40]. In addition, CXCR7 can modulate CXCR4-mediated responses by serving as a scavenger [41], [42], [43], [44] of CXCL12 or by forming heterodimers [45], [46] with CXCR4. Recently, it was reported that the C-terminal domain of CXCR7 regulates the scavenger/heterodimerization function of CXCR7. Since CXCR4+CXCR7+ (double positive), and two single positive subpopulations, i.e. CXCR4+CXCR7− and CXCR4−CXCR7+, are present in all GBM cell lines we studied, it is possible that in GBM, CXCR7 and CXCR4 not only activate their own distinct signaling pathways, but also interact with each other to create a diverse response to CXCL12 stimulation.

Despite great heterogeneity of expression and functional responses of CXCR4 and CXCR7 in these human GBM lines, we also found a consistent increase of CXCR4, CXCR7, and CXCR3 in the slow cycling subpopulation of GBM cells. The slow cycling subpopulation has a higher tumor initiating capacity and is enriched in stem cell-like markers [28]. CXCR4 has been shown to be increased in CD133+ glioma stem cells [47], while CXCR7 marks the bulk population of glioma cells [25]. Of the four lines under study, only one line (L0) displayed CXCL12 regulated in vitro sphere formation. Published data would have predicted that CXCR4 was responsible for this activity but our pharmacological analysis using CXCR4 and CXCR7 antagonists indicated that CXCR7 controls in vitro sphere formation in this line. However, results from experiments examining the potential of CXCR4− or CXCR7-expressing subpopulations to establish tumors in vivo, indicated that tumor formation is independent of the presence of CXCR4 or CXCR7. Based on the results of Hattermann et al. [25] we also anticipated that stimulation of CXCR7 would have protected GBM cells from a TMZ apoptotic insult. However, while all lines were sensitive to TMZ-induced apoptosis, none were protected from apoptosis by CXCL12. This lack of a CXCR7 effect to inhibit apoptosis is likely related to the nature of the cell culture system used to evaluate this phenomenon. The CXCR7 phenotype reported previously was found in adherent GBM cells grown in serum, which is distinct from studies reported here that were performed on sphere cultures, i.e. non-adherent serum-free conditions. In fact, data from Hattermann and colleagues indicated that CXCR7 stimulation had little to no effect on camptothecin-induced apoptosis in a glioblastoma stem-like cell line. These authors attributed the lack of effect as a result of low level expression of CXCR7 in the stem-like cells.

In summary, CXCR4 and CXCR7 are expressed in a very heterogenous manner by primary patient-derived GBM cells in vitro, which did not show up in RNA-based analysis. Coupled with diverse and heterogenous functional responses associated with CXCR4 and CXCR7 stimulation, including regulation of cell growth, migration, sphere formation, and tube formation, our results suggest that the CXCL12-CXCR4-CXCR7 system possesses the potential for considering personalized targets for human GBM therapy.
